# Der Beitrag von epidemiologischen Modellen zur Beschreibung des Ausbruchsgeschehens der COVID-19-Pandemie

**DOI:** 10.1007/s00103-021-03390-1

**Published:** 2021-07-30

**Authors:** Viola Priesemann, Michael Meyer-Hermann, Iris Pigeot, Anita Schöbel

**Affiliations:** 1 Max-Planck-Institut für Dynamik und Selbstorganisation, Am Faßberg 17, 37077 Göttingen, Deutschland; 2 Helmholtz-Zentrum für Infektionsforschung, Braunschweig und Technische Universität Braunschweig, Braunschweig, Deutschland; 3 Leibniz-Institut für Präventionsforschung und Epidemiologie – BIPS, Bremen, Deutschland; 4 Fachbereich Mathematik und Informatik, Universität Bremen, Bremen, Deutschland; 5 Fraunhofer-Institut für Techno- und Wirtschaftsmathematik ITWM, Kaiserslautern, Deutschland; 6 Fachbereich Mathematik, Technische Universität Kaiserslautern, Kaiserslautern, Deutschland

**Keywords:** Agentenbasierte Modelle, Dunkelziffer, Infektionssterblichkeit, Kompartmentmodelle, Reproduktionszahl, Agent-based models, Dark figure, Infection fatality rate, Compartmental models, Reproductive number

## Abstract

Nach dem globalen Ausbruch der COVID-19-Pandemie entwickelte sich eine Infektionsdynamik von immensen Ausmaßen. Seitdem wird versucht, das Infektionsgeschehen mit zahlreichen Maßnahmen unter Kontrolle zu bringen. Das gelang im Frühjahr 2020 sehr gut, während im darauffolgenden Herbst die Anzahl der Infektionen stark anstieg. Zur Vorhersage des Infektionsgeschehens werden epidemiologische Modelle eingesetzt, die grundsätzlich ein sehr wertvolles Werkzeug im Pandemiemanagement sind. Allerdings beruhen sie teils immer noch auf Vermutungen bzgl. der Übertragungswege und möglicher Treiber der Infektionsdynamik. Trotz zahlreicher einzelner Ansätze fehlen auch noch heute in vielen Bereichen systematische epidemiologische Daten, mit denen z. B. die Wirksamkeit einzelner Maßnahmen nachgewiesen werden könnte. In Studien generierte Daten werden aber benötigt, um möglichst belastbare Vorhersagen bzgl. des weiteren Verlaufs der Pandemie treffen zu können. Dabei entwickelt sich die Komplexität der Modelle Hand in Hand mit der Komplexität der zur Verfügung stehenden Daten. In diesem Artikel wird nach einer Abgrenzung zweier grundsätzlicher Modellklassen der Beitrag epidemiologischer Modelle zur Beurteilung verschiedener zentraler Aspekte des Pandemieverlaufs, wie z. B. Reproduktionszahl, Dunkelziffer, Infektionssterblichkeit, sowie zur Berücksichtigung der Regionalität aufgezeigt. Anschließend wird der Einsatz der Modelle zur Quantifizierung der Wirkung von Maßnahmen und der Effekte der Strategie des Testens, Nachverfolgens und Isolierens („test-trace-isolate strategy“) beschrieben. In der abschließenden Diskussion werden die Limitationen solcher Modellierungsansätze ihren Vorteilen gegenübergestellt.

## Abstract (en)

After the global outbreak of the COVID-19 pandemic, an infection dynamic of immense extent developed. Since then, numerous measures have been taken to bring the infection under control. This was very successful in the spring of 2020, while the number of infections rose sharply the following autumn. To predict the occurrence of infections, epidemiological models are used. These are in principle a very valuable tool in pandemic management. However, they still partly need to be based on assumptions regarding the transmission routes and possible drivers of the infection dynamics. Despite numerous individual approaches, systematic epidemiological data are still lacking with which, for example, the effectiveness of individual measures could be quantified. Such information generated in studies is needed to enable reliable predictions regarding the further course of the pandemic. Thereby, the complexity of the models could develop hand in hand with the complexity of the available data. In this article, after delineating two basic classes of models, the contribution of epidemiological models to the assessment of various central aspects of the pandemic, such as the reproduction rate, the number of unreported cases, infection fatality rate, and the consideration of regionality, is shown. Subsequently, the use of the models to quantify the impact of measures and the effects of the “test–trace–isolate” strategy is described. In the concluding discussion, the limitations of such modelling approaches are juxtaposed with their advantages.

## Einleitung

Nach dem globalen Ausbruch der COVID-19-Pandemie Ende 2019 und der Meldung des ersten Falls in Deutschland am 27.01.2020 entwickelte sich eine Infektionsdynamik von immensen Ausmaßen. Im März 2020 wurden in Deutschland zahlreiche Maßnahmen beschlossen, um das Infektionsgeschehen unter Kontrolle zu bringen. Auch wenn zu diesem Zeitpunkt bestimmte Eigenschaften des Virus bereits mehr oder weniger bekannt waren, beruhten die Maßnahmen zum Teil auf Vermutungen bzgl. möglicher Treiber der Infektionsdynamik und der Übertragungswege, da die Datenlage nicht ausreichend war, um eine bessere Grundlage für evidenzbasierte Entscheidungen zu bekommen. Noch heute, im Frühjahr 2021, fehlen in vielen Bereichen systematische epidemiologische Studien (s. auch [1]), mit denen z. B. die Wirksamkeit einzelner Maßnahmen nachgewiesen werden könnte.

Mathematisch-statistische Modelle benötigen verlässliche Informationen zum Infektionsgeschehen, um möglichst belastbare Vorhersagen bzgl. des weiteren Verlaufs der Pandemie treffen zu können. Die Komplexität der Modelle kann sich daher nur Hand in Hand mit der Komplexität der zur Verfügung stehenden Daten entwickeln. Die Modelle waren und sind ein wertvolles Werkzeug im Pandemiemanagement, da sie zur Abschätzung von zukünftigen Entwicklungen der Pandemie eingesetzt werden können. So bieten epidemiologische Modelle die Möglichkeit, unter Ausnutzung von vorliegenden Daten bestimmte Szenarien z. B. bezüglich der Auswirkungen des Lockdowns oder einzelner Eindämmungsmaßnahmen „durchspielen“ zu können.

In den folgenden Abschnitten wird nach einer Abgrenzung zweier grundsätzlicher Modellklassen, der sogenannten Kompartmentmodelle und der agentenbasierten Modelle, der Beitrag epidemiologischer Modelle zur Beurteilung verschiedener zentraler Aspekte des Pandemieverlaufs, wie z. B. Reproduktionszahl, Dunkelziffer und Infektionssterblichkeit, sowie zur Berücksichtigung der Regionalität aufgezeigt. Anschließend wird der Einsatz der Modelle zur Quantifizierung der Wirkung von Maßnahmen und der Effekte der Strategie des Testens, Nachverfolgens und Isolierens („test-trace-isolate strategy“) beschrieben. In der abschließenden Diskussion werden die Limitationen solcher Modellierungsansätze ihren Vorteilen gegenübergestellt.

## Modellierung der COVID-19-Pandemie

**Figure 1:**
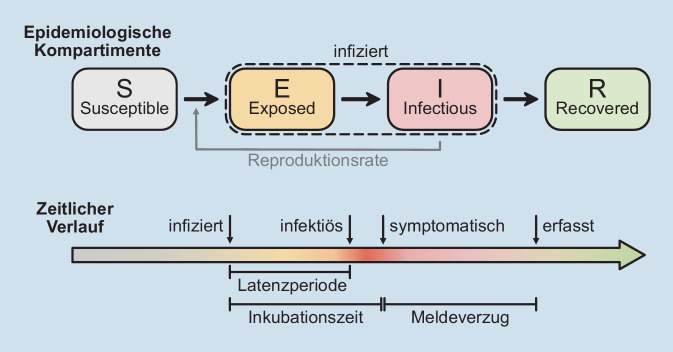

Die Dynamik einer Pandemie kann mit mathematischen Modellen beschrieben werden. Der klassische Ansatz des Kompartmentmodells basiert auf Differenzialgleichungen, in denen die Menschen nicht individuell, sondern als Menge beschrieben werden, die sogenannten SIR- und SEIR-Modelle ([2, 3, Abb. 1]).

Diese Abkürzungen stehen für 4 verschiedene Krankheitsstufen: Die Suszeptiblen (S) sind empfänglich für das Virus, die Exponierten (E) tragen es, die Infektiösen (I) geben es weiter und die Genesenen („recovered“, R) tragen nicht mehr zur Ausbreitung bei, weil sie z. B. immun sind. In die Gleichungen setzt man dann die Übergangsraten von einer in die nächste Stufe ein. Diese Modelle sind gut geeignet, um größere Strukturen, wie zum Beispiel Deutschland als Ganzes, zu betrachten. In solchen Kontexten beziehen sie sich auf hinreichend viele Menschen, sodass die gemachte Näherung gut zu rechtfertigen ist. Allerdings gehen reale Kontakte hier nur phänomenologisch ein: Da die Menschen in dem Modell nicht einzeln abgebildet sind, können sie auch keine echten Kontakte haben. Dafür ist dieses Modell analytisch lösbar und braucht wenig Rechenkapazität.

Alternativ können reale Kontaktdaten auch verwendet werden, um sogenannte agentenbasierte Modelle (ABM) zu entwickeln [4]. In ABM entspricht jeder Agent einer Person. Es werden Knoten definiert und zu einem Netzwerk verbunden. Solche Knoten stellen zum Beispiel Wohnungen, Supermärkte, Geschäfte, Schulen, Arbeitsplätze, Krankenhäuser und Kulturstätten dar, also Orte, an denen sich Menschen aufhalten können. Jeder Agent befindet sich immer in einem der Knoten und kann sich von Knoten zu Knoten bewegen. Diese Bewegungen werden nach Tageszeit aufgelöst: Eine Person ist z. B. morgens zu Hause, geht dann zum Knoten Arbeitsplatz, abends zum Knoten Supermarkt und ist dann wieder zu Hause. Die Haushalte werden in der Personenzahl und der Alterszusammensetzung so gewählt, wie es der beschriebenen Region entspricht. Diese tägliche Bewegung der Agenten zwischen den Knoten erzeugt Kontakte zwischen den Agenten. Die Zahl der Kontakte wird für jeden Knoten spezifisch aus gemessenen Kontaktdaten abgeleitet [5]. Damit ist dem Modell nicht nur jeder Kontakt bekannt, sondern auch der Ort und die Zeit des Kontakts.

Um in ABM Infektionen zu beschreiben, wird jedem Agenten ein Gesundheitszustand zugeordnet. Jeder Agent durchlebt die Infektionen, so wie es die öffentlich zugänglichen Daten über den Infektionsverlauf vorgeben. Insbesondere wird der Krankheitsverlauf altersabhängig „ausgewürfelt“. Das führt auch zu verändertem Verhalten in dem Sinne, dass ein kranker Agent z. B. den Knoten Arbeitsplatz nicht aufsuchen wird oder dass sich Agenten anstecken, bevor Symptome aufgetreten sind. Da sich mit SARS-CoV‑2 Infizierte bereits vor Ausbruch der Symptome ansteckend sein können, sind auch Agenten im Modell ohne Symptome bereits ansteckend. Wenn diese verschiedene Knoten aufsuchen und Kontakte haben, entstehen im Modell Infektionsketten. Bei einer identifizierten Infektion wird der zugehörige Agent zu Hause isoliert und es ist möglich, mit beliebiger Präzision die Kontakte der erkrankten Person zurückzuverfolgen, da in der ABM-Simulation jeder Kontakt bekannt ist.

## Grundlegende Aspekte der Modellierung

### Berechnung der Reproduktionszahl R

Die effektive Reproduktionszahl, umgangssprachlich *R*-Wert, quantifiziert, wie viele Menschen von einer infizierten Person im Mittel angesteckt werden. Im Einzelfall kann das keine/r sein oder auch sehr viele. Für die Ausbreitung ist vornehmlich der Mittelwert wichtig: Ist *R* effektiv kleiner als 1, dann gehen die Fallzahlen exponentiell zurück, ist *R* größer als 1, dann wachsen sie exponentiell. Wie schnell sie wachsen oder zurückgehen, hängt sowohl vom genauen *R*-Wert ab als auch von der Generationszeit. Das kann man sich leicht verdeutlichen: Startet man mit 100 Infizierten in einer sehr großen Population, dann stecken diese im Mittel nach einer Generationszeit R * 100 Menschen an. Nach *k* Generationen erhält man also *R*^*k*^-mal mehr Infizierte. Ein solches Wachstum wird durch eine Exponentialfunktion beschrieben: Diese wächst exponentiell, wenn *R* größer als 1 ist, und geht exponentiell zurück, wenn *R* kleiner als 1 ist. Wenn ein relevanter Anteil der Bevölkerung immun ist, dann geht der effektive *R*-Wert zurück.

**Figure 2:**
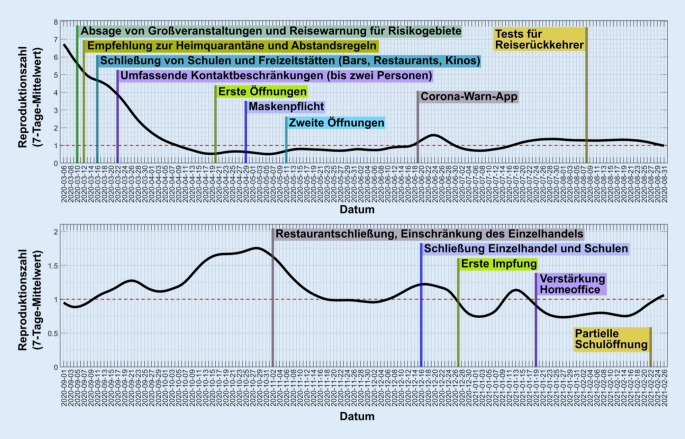

Die effektive Reproduktionszahl *R* kann man grob abschätzen, indem man die Anzahl der Neuinfektionen *N(t)* zu einem Zeitpunkt *t* durch die Anzahl 4 Tage vorher dividiert: $\hat{R}=N\left(t\right)/N\left(t-4\right)$. Diese 4 Tage entsprechen der vom Robert Koch-Institut (RKI) angenommenen Generationszeit von SARS-CoV‑2. Um statistische Schwankungen zu glätten, kann man entweder über mehrere Tage den Mittelwert berechnen [6] oder ein Inferenzmodell nutzen [3, 7, 8]. In der einfachsten Rechnung nimmt man an, dass der Eintrag neuer Fälle von außen, *H(t), *sehr gering ist. Nimmt man dagegen an, dass dieser Eintrag je Generationszeit relevant ist, dann sollte er auf geeignete Weise in die Berechnung einfließen, denn dann ergibt sich die Fallzahl *N(t)* als $N\left(t\right)=R*N\left(t-4\right)+H\left(t\right)$ und der *R*-Wert somit als $\hat{R}=\left(N\left(t\right)-H\left(t\right)\right)/N\left(t-4\right)$. Alternativ, kann der *R*-Wert aus SEIR-Modellen analytisch berechnet werden [9]. Die Entwicklung des *R*-Werts von dem Beginn der Pandemie bis Ende Februar 2021 ist in Abb. 2 dargestellt.

Im Gegensatz zum effektiven *R*-Wert, der vom Anteil immuner Personen und vom Verhalten der Bevölkerung abhängt, gibt die Basisreproduktionszahl *R*_*0*_ an, wie stark sich das Virus ausbreitet, wenn eine Bevölkerung sich „normal“ verhält und kein Mitglied gegenüber dem Virus immun ist. Da es eine solche Normbevölkerung nicht gibt, variiert dieser Wert von Region zu Region und kann nur mit einer gewissen Unsicherheit bestimmt werden. Für Europa wird typischerweise bei der ursprünglichen Variante von SARS-CoV‑2 von einem *R*_*0*_ von 2,8–3,8 ausgegangen [10].

### Auswirkung der Dunkelziffer

Gerade bei COVID-19 verläuft ein nennenswerter Teil der Infektionen (fast) symptomlos oder nur mit sehr leichten Symptomen, sodass infizierte Personen leicht übersehen werden können. Es ist daher anzunehmen, dass die Anzahl der mit SARS-CoV‑2 Infizierten die gemeldeten Fallzahlen deutlich übersteigt. Diese sogenannte Dunkelziffer gibt an, mit welchem Faktor man die gemeldeten Fallzahlen multiplizieren muss, um die tatsächlichen Fallzahlen zu erhalten. Neben Antikörpertests aus Blutproben kann man eine Untergrenze für die Dunkelziffer auch mit mathematischen Modellen basierend auf altersabhängigen Prävalenzen schätzen [11]. Dennoch bleibt die Dunkelziffer eine Unbekannte.

Sind nun alle Ergebnisse der epidemiologischen Modelle wegen der fehlenden Dunkelziffer mit großer Vorsicht zu genießen? Das ist erfreulicherweise nicht der Fall, da die Dunkelziffer nur dann eine Auswirkung auf die Simulationen hat, wenn sich ein Großteil der Bevölkerung schon infiziert hat. Dazu betrachten wir zwei konkrete Fragestellungen anhand eines Beispiels genauer. Es geht um die Schätzung des weiteren Pandemieverlaufes am 19.10.2020, zu Beginn der Herbstwelle. An diesem Tag hielt die Bundeskanzlerin im Fernsehen eine Rede, in der sie die Bevölkerung bat, vorsichtig zu sein. Rückblickend wissen wir, dass u. a. diese Rede und die nachfolgenden Lockdowns zu Verhaltensänderungen führten. Wir nutzen diesen Tag jetzt, um zu illustrieren, was passiert wäre, wenn wir unser Verhalten nicht geändert hätten, und welche Auswirkung die Dunkelziffer dabei hätte.

**Figure 3:**
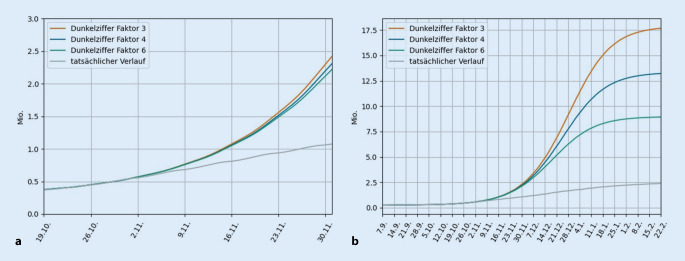

Dazu werden in Abb. 3 die drei Dunkelziffern 3, 4 und 6 betrachtet, also dass sich 3‑, 4‑ oder 6‑mal mehr Personen infizieren, als man entdeckt. Die graue Linie zeigt zum Vergleich den tatsächlichen Infektionsverlauf, bei dem wir unser Verhalten geändert hatten.

- In der Vergrößerung auf der linken Seite der Abbildung sieht man, dass die Vorhersage am 19.10-2020 für die nächsten Wochen von der Dunkelziffer nur wenig beeinflusst wird: Die drei Kurven für die Dunkelziffern liegen dicht beieinander. Das gilt so auch für andere Verhaltensannahmen.
- Dagegen hat die Dunkelziffer große Auswirkungen, wenn man den Verlauf der Pandemie bis zu ihrem Ende bedingt durch Populationsimmunität oder endemische Ausbreitung simuliert. Die Abbildung zeigt, dass sich die drei Kurven immer unterschiedlicher verhalten, je mehr Personen (unentdeckt) infiziert wurden. Die Kurven werden konstant, wenn eine Populationsimmunität („Herdenimmunität“) erreicht ist, sodass das Virus sich nicht mehr weiter ausbreiten kann. Als Faustregel gilt, dass der Anteil der immunisierten Personen mindestens 1‑1/*R*_*0*_ betragen muss, wobei *R*_*0*_ die oben genannte Basisreproduktionszahl der Krankheit ist. Für *R*_*0*_ = 3 ergibt sich, dass mindestens 2/3 der Bevölkerung Antikörper aufbauen müssen. Für eine Dunkelziffer von 3 ist dieser Zustand erreicht, wenn man ca. 18 Mio. Infizierte entdeckt hat, bei einer Dunkelziffer von 4, wenn man 13,5 Mio. Infizierte entdeckt hat, und bei einer Dunkelziffer von 6 sogar schon, wenn man 9 Mio. Infizierte entdeckt hat.

### Einfluss des Alters auf Sterberaten und Infektionsdynamik

Die Wahrscheinlichkeit, an einer Infektion mit COVID-19 zu versterben, ist stark vom Alter der Infizierten abhängig. Dabei unterscheidet man zwischen der Fallsterblichkeit („case fatality rate“ [CFR]), die das Verhältnis von Todesfällen zu gemeldeten Krankheitsfällen beschreibt, und der Infektionssterblichkeit („infection fatality rate“ [IFR]), die das Verhältnis von Todesfällen zur Anzahl der Infizierten angibt. Die beiden Werte unterscheiden sich also durch die Dunkelziffer, die bei der Berechnung der Infektionssterblichkeit mit einbezogen wird. Studien zeigen [12–14], dass sich die Infektionssterblichkeit mit jeweils zusätzlichen 20 Lebensjahren etwa verzehnfacht. Das folgende Beispiel illustriert, wie drastisch die Sterbewahrscheinlichkeit im Alter zunimmt: Liegt die Infektionssterblichkeit für eine Person mit 40 Jahren also zum Beispiel bei rund 0,05 %, sind es im Alter von 60 Jahren schon 0,5 % und im Alter von 80 Jahren sogar 5 %. Diese Verzehnfachung zeigt sich auch anhand der RKI-Daten (siehe [13]). Bei der Schätzung der Todesfälle muss daher die Altersverteilung der SARS-CoV-2-Infektionen unbedingt berücksichtigt werden. Das klingt einfach, ist es aber nicht, da sich diese Verteilung dynamisch ändern kann. Das hat sich im Jahr 2020 in Deutschland gezeigt: Im Sommer waren vor allem jüngere Personen von der Infektion betroffen, sodass die Fallsterblichkeit bei unter 0,3 % (z. B. Kalenderwoche 36) lag. In der zweiten Welle im Winter waren die Älteren deutlich stärker betroffen; die Fallsterblichkeit stieg auf rund 4 % (Kalenderwoche 53; [15]).

Die Alterskohorten sind nicht nur wichtig, um die Sterblichkeiten differenziert zu berechnen, sondern auch um die weitere Ausbreitung der Pandemie abschätzen zu können. In epidemiologischen Modellen kann dazu eine (nicht zu große) Zahl interagierender Gruppen frei gewählt werden. Diese Gruppen können auch genutzt werden, um Kontaktraten besser zu modellieren: So haben Schulkinder eine größere Anzahl an Kontakten als die Gruppe der über 80-Jährigen. Pro Altersgruppe wird die Zahl der jemals Infizierten als Zeitreihe gespeichert. Unter der Annahme fester Perioden für Inkubation und infektiöse Phase lässt sich daraus pro Gruppe die Zahl der aktuell Infektiösen und der projizierten Sterbefälle berechnen und über Kontaktraten innerhalb und zwischen den Altersgruppen die Zahl der Neuinfektionen besser abschätzen. Diese werden mit den aktuell gemeldeten Zahlen verglichen, sodass die Modellparameter im Laufe der Simulation angepasst werden können.

### Berücksichtigung der Regionalität

Die Regionalität ist in einer Pandemie ein wesentlicher Aspekt. Jede Region weist eine eigene Infrastruktur auf, eine spezifische Demografie sowie Besonderheiten wie spezielle Industrien, die das Infektionsgeschehen beeinflussen. Daher ist es für das Verständnis der Pandemie wichtig, diese Besonderheiten in der Bewertung eines Ausbruchs zu berücksichtigen. Dies ist insbesondere dann wichtig, wenn die Pandemie in unterschiedlichen Regionen unterschiedlich stark verläuft. Kompartmentmodelle und ABM erlauben eine regionale Betrachtung. Es ist naheliegend, dass man sich mit SIR-Modellen weniger auf die Besonderheiten der Region selbst, sondern eher auf die Beschreibung von Bewegungen zwischen Regionen fokussiert [16]. In ABM kann man die Besonderheiten der Region durch die Auswahl und die Belegung der Knoten kleinräumig bzw. den lokalen Gegebenheiten angepasst abbilden, wie z. B. die lokal vorhandene Zahl von Supermärkten und Schulen bei gegebener Zahl der Einwohner sowie deren Demografie. So kann die Abhängigkeit der Pandemievorhersagen von den lokalen Spezifika untersucht werden: Wie wirkt sich etwa ein stärkeres Zusammenleben von verschiedenen Generationen im gleichen Haushalt in einer Region auf die Pandemie und auf die Sterbezahlen aus? Wie wirkt sich ein großer lokaler Arbeitgeber auf das Pandemiegeschehen aus? Was sind die besten Maßnahmen, wenn sich lokal ein Ausbruch in einem solchen großen Betrieb entwickelt im Vergleich zu kleinen verteilten Infektionsgeschehen? All diese Fragen können mit ABM, die hinreichend genau auf die jeweils betroffene Region angepasst wurden, beantwortet werden. Die so an die jeweilige Region angepassten Simulationen können helfen, effiziente und angemessene Maßnahmen zur Eindämmung von Infektionsausbrüchen zu empfehlen.

## Quantifizierung von Eindämmungsmaßnahmen

### Abschätzung der Wirkung von Eindämmungsmaßnahmen

**Figure 4:**
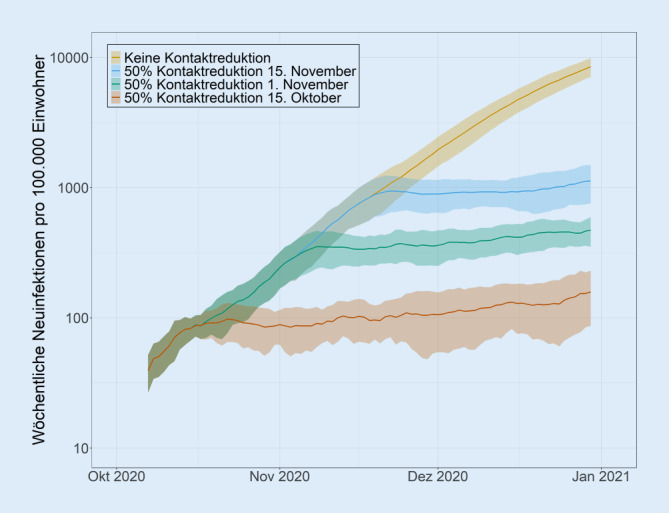

Solange nicht ausreichend Impfstoff oder effektive Behandlungen für COVID-19 bereitstehen, kann die Ausbreitung durch nichtpharmakologische Interventionen (NPI) verlangsamt werden. Die NPI richten ihre Wirkung darauf, die Übertragung des Virus bei einem Kontakt weniger wahrscheinlich zu machen und insgesamt die Anzahl der Kontakte zu reduzieren (Abb. 4). Dazu gehören also auf der einen Seite die AHA + LA-GGG-Maßnahmen (Achten auf Abstand, Hygiene, Alltagsmaske + Lüften und Verwendung der App; Vermeiden von geschlossenen Räumen, Gruppen und Gedränge sowie lebhaften Gesprächen dicht an dicht) und auf der anderen Seite konkrete Verordnungen, wie das Schließen von Restaurants, Bildungseinrichtungen, Einzelhandel, Nutzen des Homeoffice, wo es möglich ist, sowie Kontaktbeschränkungen im Privaten. Das Ziel ist, mit einer Kombination dieser NPI den effektiven *R*-Wert auf oder unter 1 zu bringen.

Wie schätzt man nun die Wirkung von NPI aus Daten? In einem kontrollierten Labor- oder Feldexperiment würde man eine NPI randomisiert anwenden und die Wirkung mit Kontrollexperimenten oder -gruppen vergleichen. Aufgrund der Dringlichkeit bei COVID-19 wurden solche Studien nicht realisiert. Deswegen nutzen wissenschaftliche Analysen den Vorteil sogenannter natürlicher Experimente, bei denen die Probanden aufgrund von ungeplanten, natürlichen Ereignissen in Experimentalgruppe und Kontrollgruppe eingeteilt werden: Verschiedene Länder weltweit haben verschiedene Kombinationen von NPI zu verschiedenen Zeiten eingesetzt [7, 8, 17, 18]. Welche Länder welche NPI genutzt haben, findet sich z. B. in [19, 20].

Start und Ende dieser NPI setzt man dann zur Entwicklung der täglichen Fallzahlen ins Verhältnis (Infektions‑, Krankenhaus- oder Sterbezahlen). In einer solchen Analyse werden also die Zeitspanne der verschiedenen NPI sowie die Dynamik der Ansteckung (Inkubationsperiode, Meldeverzögerung etc.) berücksichtigt. Um dann die Wirkung der NPI abzuschätzen, verwendet man Bayesianische Inferenzverfahren, die viele Varianten eines SIR- oder SEIR-Modells und der NPI-Stärken durchspielen. Als Ergebnis erhält man dann die Parameter, die die Daten am besten erklären, sowie deren Glaubwürdigkeitsintervalle. Man kann sich dieses Vorgehen als eine komplexe Erweiterung klassischer Regressionsmethoden vorstellen.

Anstatt die Wirkung der NPI aus den Fallzahlen abzuschätzen, können auch andere Daten, wie z. B. Bewegungsdaten und Aufenthaltsorte, genutzt werden. Die Mobiltelefonbewegungsdaten wurden z. B. in einer US-amerikanischen Studie genutzt, um zu untersuchen, wann sich Personen wie lange an bestimmten Orten aufgehalten haben [21]. Als Ergebnis konnte man abschätzen, dass insbesondere Ansteckungen in Restaurants, in Fitnessstudios, Cafés, Hotels und bei religiösen Veranstaltungen stattfanden. Nimmt man den gesamten Einzelhandel zusammen, dann trug er ebenso viel bei. Schulen konnten in diesem Zusammenhang nicht explizit untersucht werden, da insbesondere jüngere Schüler nicht alle über Mobiltelefone verfügten. Schulen sind allerdings in einem anderen Kontext aufgefallen: Im Vereinigten Königreich gab es im November 2020 einen umfassenden Lockdown, jedoch blieben die Schulen offen. Gleichzeitig wurden im Land jede Woche rund 100.000 Zufallstests durchgeführt, sodass ein objektiver Datensatz zur Krankheitsausbreitung in allen Altersgruppen vorlag. Dabei zeigte sich, dass der Anteil positiver Tests in der gesamten Bevölkerung bei rund 1 % lag, während er bei Schülern 2 % und sogar höher war [22]. Nach der Schulschließung hat sich dieser Anteil an den der anderen Altersklassen wieder angepasst [23]. Das ist ein klarer Hinweis auf Ansteckungen in den Schulen und darauf, dass Schulen die Treiber der Ausbreitung werden, wenn alle anderen Bereiche geschlossen sind. Sind andere Bereiche jedoch offen, dann scheinen Schulen etwa ebenso viel wie andere Bereiche zur Ausbreitung beizutragen [24].

Speziell für Deutschland berichtet das Robert Koch-Institut, in welchem Kontext Ansteckungen vermutlich stattgefunden haben. Solche Datensätze sind ebenfalls informativ, jedoch muss man beachten, dass diese auf subjektiven Berichten beruhen und nur begrenzt verallgemeinert werden können.

Wie wirksam ist nun jede einzelne Maßnahme? Nimmt man viele Studien zusammen, dann zeigt sich, dass jedes der größeren Maßnahmenpakete (Verbot von größeren Treffen und Veranstaltungen, Schließen von Schulen, Universitäten und Betreuungseinrichtungen, Homeoffice, Schließen von Restaurants und Einzelhandel, Kontaktbeschränkungen, AHA + LA-GGG-Maßnahmen und effizientes Testen) jeweils zwischen 10 % und 40 % Reduktion des *R*-Wertes bringt; bei strikter Umsetzung der Maßnahmen kann eine größere Reduktion erreicht werden. Doch selbst wenn die Wirkung aus vergangenen Daten recht gut geschätzt werden kann, wird sie bei einer zukünftigen Implementierung immer von der genauen Umsetzung abhängen sowie von der Motivation der Bevölkerung und den konkreten Hygienemaßnahmen. Um das Beispiel „Schulen“ zu nennen: Werden Masken getragen und wird regelmäßig gelüftet? Gibt es kleine, feste Gruppen oder wechselnde Kontakte? Bleiben Schüler und Lehrkräfte bei Erkältungssymptomen oder COVID-19-Verdacht zu Hause? Wird präventiv getestet und werden Kontakte effektiv nachverfolgt? All das beeinflusst die Ansteckungswahrscheinlichkeiten, sodass die genaue Auswirkung eines Maßnahmenpakets nur schwer vorherzusagen ist.

**Figure 5:**
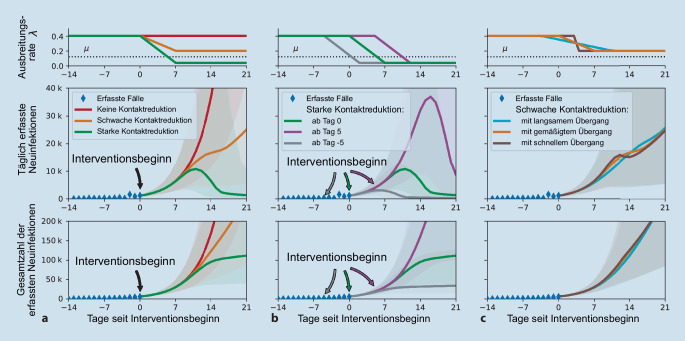

Generell gilt gerade für das steile Wachstum einer Welle: Je früher man eine Maßnahme umsetzt, desto (deutlich) mehr Infektionen und Todesfälle werden vermieden. Am Beispiel der ersten Welle zeigt sich klar, dass schon fünf Tage einen immensen Einfluss haben können (Abb. 4 und 5). Ebenso wichtig ist es, dass die Maßnahmen ausreichend wirksam sind. Ansonsten verlangsamen sie den Anstieg nur, können ihn aber nicht zu einem Rückgang umkehren (Abb. 5).

### Abschätzung der Wirkung von Maßnahmen mit agentenbasierten Modellen

Will man die Wirkung von Maßnahmen mit einem ABM abschätzen, so muss man gewisse Parameter, die nicht aus den realen Kontaktdaten und demografischen Daten abzulesen sind, an die reale Entwicklung der Pandemie anpassen. Entscheidend ist die Übertragungswahrscheinlichkeit bei einem Kontakt zwischen einem gesunden und einem kranken Agenten in einem Knoten. Diese Wahrscheinlichkeit hängt direkt mit der Reproduktionszahl zusammen und wird an Daten aus einer Phase einer relativ ungestörten exponentiellen Ausbreitung des Infektionsgeschehens angepasst (wie in Abb. 4 geschehen). Damit ist ein gut definierter Ausgangspunkt des ABM erstellt, der es erlaubt, die Auswirkung verschiedener Maßnahmen abzuschätzen.

So ist es etwa möglich, eine Schulschließung zu simulieren, wodurch die Kontakte in dem Knoten „Schule“ auf null gesetzt werden. Durch Wiederholung von Simulationen mit und ohne Schulschließung kann man so deren Auswirkung auf das epidemiologische Geschehen abschätzen [25]. Beide Szenarien müssen hinreichend oft simuliert werden, um einen Mittelwert des zu erwartenden Verlaufs mit und ohne Schulschließung sowie eine Fehlerbreite zu erhalten, die als Konfidenz in die Vorhersage interpretiert werden kann.

Es ist bei der Bewertung solcher Vorhersagen immer zu beachten, dass die Annahmen über die Kontakte in den verschiedenen Knoten sowie die Übertragungswahrscheinlichkeit pro Kontakt entscheidenden Einfluss auf die Vorhersage haben. So macht die Unsicherheit über die tatsächliche Infektiosität von kleinen Kindern auch die Vorhersage über den Effekt von Schul- und Kitaschließungen unsicher. Die Vorhersagen aus dem ABM sind einerseits extrem verlässlich, weil sie die Prozesse in der Gesellschaft sehr gut abbilden können. Sie können andererseits aber nie sicherer werden als das Wissen über Übertragungswege und Kontakte, das in die ABM eingeht.

### Quantifizierung des Effekts der Test-Trace-Isolate-Strategie

Ein effektiver Beitrag zur Eindämmung einer Pandemie sind (i) das Testen, (ii) das Isolieren infizierter Personen und (iii) die Kontaktnachverfolgung und Quarantäne, um Infektionsketten zu stoppen [26, 27]. Diese Maßnahmen sind besonders dann effektiv, wenn die Kontaktpersonen identifiziert werden, bevor sie weitere Personen angesteckt haben. Damit die Kontaktnachverfolgung schnell genug sein kann, dürfen die Gesundheitsämter nicht überlastet sein. Sind diese erst einmal überlastet, dann werden Kontaktpersonen nicht schnell genug quarantänisiert und haben bereits weitere Personen angesteckt. Je niedriger die Fallzahlen sind, desto effektiver kann diese Eindämmungsstrategie umgesetzt werden. Mathematisch führt die limitierte Kapazität zu einem Kipppunkt: Werden die Fallzahlen zu hoch, kommt es zu einer sich selbst verstärkenden Ausbreitung [13, 26]. Sind hingegen die Fallzahlen niedrig, dann bringt die Kontaktnachverfolgung zusätzliche Stabilisierung. Aus epidemiologischer Sicht ist es also unstrittig: Eine Pandemie lässt sich rein technisch bei niedriger Inzidenz leichter eindämmen und jede Person hat mehr Freiheiten [28].

## Diskussion

Die oben vorgestellten Modelle waren zum Teil in geradezu beeindruckender Weise in der Lage, das Infektionsgeschehen vorherzusagen. Allerdings sind sie auch des Öfteren an ihre Grenzen gestoßen. So kann der Wert von *R* zu einem bestimmten Zeitpunkt erst nach einer Verzögerung von 2–3 Wochen mit einer angemessenen Sicherheit geschätzt werden. Diese Verzögerung ergibt sich u. a. aus einer Kombination verschiedener Faktoren wie Inkubationszeit, Zeit bis zur Testung, Auswertung und Veröffentlichung des Testresultats sowie notwendige Zeitspanne zur Ansammlung von Evidenz aus den beobachteten Daten. Wegen dieser Verzögerung können sich auch die Auswirkungen von verordneten Eindämmungsmaßnahmen oder von Lockerungen erst mit beträchtlicher Verspätung in den gemeldeten Fallzahlen zeigen, was z. B. bei der Modellierung und der Bewertung der Wirksamkeit eines jeden Maßnahmenpakets berücksichtigt werden muss.

Zudem ist die Datenlage weiterhin unzureichend: Um die Wirksamkeit der verschiedenen Maßnahmen adäquat in Modellen abbilden zu können, hätte ein flächendeckendes Surveillance-System aufgesetzt werden müssen, mit dem gemäß klarer Vorgaben Daten zur Wirksamkeit der einzelnen Maßnahmen erhoben worden wären. Auf diese Weise hätte man z. B. aus dem natürlichen Experiment der Schulöffnungen Evidenz bzgl. der bestmöglichen Strategie generieren können. Noch zielführender wäre es gewesen, eine kontrollierte randomisierte Studie aufzusetzen, bei der zufällig ausgewählte Schulen verschiedene Strategien der Öffnung umgesetzt und so die für eine Entscheidungsfindung notwendige Datenlage erzeugt hätten. Ein ähnliches Vorgehen – sei es als geplantes oder als natürliches Experiment – wäre auch in anderen Bereichen womöglich sinnvoll umsetzbar gewesen. So hätte man sogar den Föderalismus aktiv nutzen können, um verschiedene Strategien zur COVID-19-Eindämmung zu erproben. Zumindest in der 2. oder 3. Welle hätten sorgfältig geplante Fallkontrollstudien aufgesetzt werden sollen, um einerseits die Ätiologie und den Verlauf der Erkrankung sowie andererseits deren Verbreitungswege besser verstehen zu können [1]. Ein solches Zusammenspiel von Wissenschaft und Gesellschaft hätte helfen können, Maßnahmen dort einzusetzen, wo sie eine besonders hohe Wirksamkeit entfalten.

Letztendlich ist damit auch klar, dass die vorgestellten Modelle nicht in der Lage sind, spezifische, kurzfristige Änderungen abzubilden und daraus Vorhersagen abzuleiten. Dies betrifft etwa das Auftreten von neuen Varianten des Virus mit höheren Infektionsraten, aber auch das menschliche Verhalten, das den Grad der Umsetzung von Maßnahmen bestimmt. Ebenso schwierig ist es, langfristige Perspektiven verlässlich zu beschreiben. Im Gegensatz dazu sind kontinuierliche Änderungen der zentralen Parameter des Infektionsgeschehens sehr gut in solchen Modellen abbildbar, solange ein Vergleich mit den tatsächlichen Daten möglich ist.

Trotz dieser Einschränkungen helfen die vorgestellten Modelle, die Auswirkungen von Maßnahmenpaketen oder Lockerungen vorauszusagen. Sie erlauben zudem, durch unterschiedliche Herangehensweisen verschiedene Perspektiven zu beleuchten und dadurch zu einer besseren Gesamteinschätzung zu gelangen [3, 29–31]. Sie zwingen Wissenschaftler:innen, die relevanten Faktoren und Kenngrößen sowie die Unsicherheiten genau zu formulieren, und tragen dadurch zu einem stringenten Durchdenken eines Sachverhalts bei. Dadurch können idealerweise quantitative Aussagen über die Wirksamkeit bestimmter Maßnahmen erzielt werden. Auf jeden Fall kann aber die zukünftige Entwicklung der Fallzahlen in alternativen Szenarien abgeschätzt werden. Damit liefern die Modelle einen wichtigen Baustein für die Entscheidungsfindung der Politik.

## Fazit

Mathematisch-statistische Modelle sind ein wichtiges Instrument, um in Krisensituationen die erforderliche politische Entscheidungsfindung zu unterstützen. Allerdings hängt ihre Aussagekraft stark von der Qualität der zur Verfügung stehenden Daten ab.

## References

[1] Zeeb H, Ahrens W, Haug U, Grabenhenrich L, Pigeot I (2021) Epidemiologische Ansätze zur Klärung wichtiger Forschungsfragen zu COVID-19 – eine Übersicht. Bundesgesundheitsblatt Gesundheitsforschung Gesundheitsschutz. 10.1007/s00103-021-03378-x 10.1007/s00103-021-03378-x PMC8276842 34258629

[2] Kermack WO, McKendrick AG (1927). A contribution to the mathematical theory of epidemics. Proc Roy Soc A.

[3] Dehning J, Spitzner FP, Linden MC (2020). Model-based and model-free characterization of epidemic outbreaks.

[4] Niaz Arifin SM, Madey R, Collins FH (2016). Spatial agent-based simulation modeling in public health: design, implementation, and applications for malaria epidemiology.

[5] Mossong J, Hens N, Jit M (2008). Social contacts and mixing patterns relevant to the spread of infectious diseases. PLoS Med.

[6] der Heiden AM, Hamouda O (2020). Schätzung der aktuellen Entwicklung der SARS-CoV-2-Epidemie in Deutschland – Nowcasting. Epidemiol. Bull..

[7] Dehning J, Zierenberg J, Spitzner FP (2020). Inferring change points in the spread of COVID-19 reveals the effectiveness of interventions. Science.

[8] Brauner JM, Mindermann S, Sharma M (2021). Inferring the effectiveness of government interventions against COVID-19. Science.

[9] Khailaie S, Mitra T, Bandyopadhyay A (2021). Development of the reproduction number from coronavirus SARS-CoV-2 case data in Germany and implications for political measures. BMC Med.

[10] Robert Koch-Institut (2021) Virus und Epidemiologie (Stand: 2.3.2021). https://www.rki.de/SharedDocs/FAQ/NCOV2019/FAQ_Liste_Epidemiologie.html. Zugegriffen: 6. März 2021

[11] Fiedler J, Moritz C, Schöbel A, Dreßler K, Speckert M, Feth S (2021) Ein mathematisches Modell zur Schätzung der Dunkelziffer von SARS-CoV-2-Infektionen in der Frühphase der Pandemie am Beispiel Deutschland und Italien. Bundesgesundheitsblatt Gesundheitsforschung Gesundheitsschutz. 10.1007/s00103-021-03384-z 10.1007/s00103-021-03384-z PMC8298962 34297161

[12] Levin AT, Hanage WP, Owusu-Boaitey N, Cochran KB, Walsh SP, Meyerowitz-Katz G (2020). Assessing the age specificity of infection fatality rates for COVID-19: Systematic review, meta-analysis, and public policy implications. Eur J Epidemiol.

[13] Linden M, Mohr SB, Dehning J (2020). Case numbers beyond contact tracing capacity are endangering the containment of COVID-19. Dtsch Arztebl Int.

[14] O’Driscoll M, Dos Santos GR, Wang L (2020). Age-specific mortality and immunity patterns of SARS-CoV-2. Nature.

[15] Robert Koch-Institut COVID-19-Fälle nach Meldewoche und Geschlecht sowie Anteile mit für COVID-19 relevanten Symptomen, Anteile Hospitalisierter und Verstorbener. https://www.rki.de/DE/Content/InfAZ/N/Neuartiges_Coronavirus/Daten/Klinische_Aspekte.html. Zugegriffen: 6. März 2021

[16] Kühn MJ, Abele D, Mitra T (2020). Assessment of effective mitigation and prediction of the spread of SARS-CoV-2 in Germany using demographic information and spatial resolution.

[17] Islam N, Sharp SJ, Chowell G (2020). Physical distancing interventions and incidence of coronavirus disease 2019: Natural experiment in 149 countries. BMJ.

[18] Haug N, Geyrhofer L, Londei A (2020). Ranking the effectiveness of worldwide COVID-19 government interventions. Nat Hum Behav.

[19] Our World in Data Policy responses to the Coronavirus pandemic. https://ourworldindata.org/policy-responses-covid. Zugegriffen: 6. März 2021

[20] World Health Organization COVID-19 situation in the WHO European Region. https://who.maps.arcgis.com/apps/opsdashboard/index.html#/ead3c6475654481ca51c248d52ab9c61. Zugegriffen: 6. März 2021

[21] Chang S, Pierson E, Koh PW (2021). Mobility network models of COVID-19 explain inequities and inform reopening. Nature.

[22] Office for National Statistics (2020) Coronavirus (COVID-19) infection survey, UK: 11 December 2020. https://www.ons.gov.uk/peoplepopulationandcommunity/healthandsocialcare/conditionsanddiseases/bulletins/coronaviruscovid19infectionsurveypilot/11december2020. Zugegriffen: 4. März 2021

[23] Office for National Statistics (2021) Coronavirus (COVID-19) infection survey, UK: 29 January 2021. https://www.ons.gov.uk/peoplepopulationandcommunity/healthandsocialcare/conditionsanddiseases/bulletins/coronaviruscovid19infectionsurveypilot/29january2021. Zugegriffen: 4. März 2021

[24] European Centre for Disease Prevention and Control (2020) COVID-19 in children and the role of school settings in transmission—First update. https://www.ecdc.europa.eu/sites/default/files/documents/COVID-19-in-children-and-the-role-of-school-settings-in-transmission-first-update_0.pdf. Zugegriffen: 4. März 2021

[25] VSP / Technische Universität Berlin R‑Wert-Rechner. https://covid-sim.info/r-calcs-v2/2021-01-24b. Zugegriffen: 6. März 2021

[26] Contreras S, Dehning J, Loidolt M (2021). The challenges of containing SARS-CoV-2 via test-trace-and-isolate. Nat Commun.

[27] Contreras S, Dehning J, Mohr SB, Spitzner FP, Priesemann V (2020). Low case numbers enable long-term stable pandemic control without lockdowns.

[28] Priesemann V, Balling R, Brinkmann MM (2021). An action plan for pan-European defence against new SARS-CoV-2 variants. Lancet.

[29] Mitze T, Kosfeld R, Rode J, Wälde K (2020). Face masks considerably reduce COVID-19 cases in Germany. Proc Natl Acad Sci U S A.

[30] Donsimoni J, Glawion R, Hartl T (2020). Covid-19 in Deutschland – Erklärung, Prognose und Einfluss gesundheitspolitischer Maßnahmen. Persp Wirtschaftspol.

[31] Bracher J, Wolffram D, Deuschel J (2020). Short-term forecasting of COVID-19 in Germany and Poland during the second wave—A preregistered study.

